# Cryptogamous Origin of Fever

**Published:** 1866-09

**Authors:** 


### CRYPTOGAMOUS ORIGIN OF FEVER.

A counter-claim to the alleged discovery of the cause of malarious fever by Dr. Salisbury, is set up by a French physician, to which the Boston Medical and Surgical Journal objects on the ground, that the main facts proving the cryptogamous origin of this variety of fevers were made known by Dr. Salisbury before that of Lemaire, the French investigator claiming priority.

About the year 1847, Dr. Mitchell, of Philadelphia, published his opinions in regard to the cause of certain febrile affections, which, from our recollection of the theory promulgated by him, (for we have not the pamphlet before us) ascribed the cause to cryptogamous poisoning.

We take pleasure in announcing that a valued and talented contributor for the Atlanta Medical and Surgical Journal is investigating this subject and will, perhaps in the next number, furnish our readers with some valuable suggestions on the Cryptogamii, and of their probable connection with the production of malarious or remittent and intermittent fevers.

The experiments of Dr. Salisbury, some of which have already been published in the June number of this Journal, seem to be very conclusive in many particulars. Should they be verified by others, to the extent of settling permanently this question, we will have prominently before us the great advantages derived from improvements in the modes of scientific investigations. To the microscope, and the improvements in its use, are we indebted for many valuable facts in etiology and pathology—practical facts which lead to decided improvements in the treatment of disease. Ingeniously wrought theories, made plausible by the talent of the author, and recognized by the medical world as true, for ages, may be overturned in a day, by ocular demonstrations of stubborn facts in the use of this instrument.

